# Microplate-based surface area assay for rapid phenotypic antibiotic susceptibility testing

**DOI:** 10.1038/s41598-018-35916-0

**Published:** 2019-01-18

**Authors:** Kelly Flentie, Benjamin R Spears, Felicia Chen, Nathan B Purmort, Kayla DaPonte, Emma Viveiros, Nicholas Phelan, Cicely Krebill, Alec N. Flyer, David C Hooper, David L. Smalley, Mary Jane Ferraro, Aleksandar Vacic, Eric Stern

**Affiliations:** 1 Selux Diagnostics, Inc., 56 Roland Street, Suite 206, Charlestown, MA 02129 USA; 2 Massachusetts General Hospital, 55 Fruit St, Boston, MA 02114 USA; 3 Harvard Medical School, 25 Shattuck St, Boston, MA 02115 USA; 4 American Esoteric Laboratories, 1701 Century Center Cove, Memphis, TN 38134 USA

## Abstract

Rapid delivery of proper antibiotic therapies to infectious disease patients is essential for improving patient outcomes, decreasing hospital lengths-of-stay, and combating the antibiotic resistance epidemic. Antibiotic stewardship programs are designed to address these issues by coordinating hospital efforts to rapidly deliver the most effective antibiotics for each patient, which requires bacterial identification and antimicrobial susceptibility testing (AST). Despite the clinical need for fast susceptibility testing over a wide range of antibiotics, conventional phenotypic AST requires overnight incubations, and new rapid phenotypic AST platforms restrict the number of antibiotics tested for each patient. Here, we introduce a novel approach to AST based on signal amplification of bacterial surfaces that enables phenotypic AST within 5 hours for non-fastidious bacteria. By binding bacterial surfaces, this novel method allows more accurate measurements of bacterial replication in instances where organisms filament or swell in response to antibiotic exposure. Further, as an endpoint assay performed on standard microplates, this method should enable parallel testing of more antibiotics than is currently possible with available automated systems. This technology has the potential to revolutionize clinical practice by providing rapid and accurate phenotypic AST data for virtually all available antibiotics in a single test.

## Introduction

Timely administration of targeted antibiotics, determined by phenotypic antimicrobial susceptibility testing (AST), is essential not only for infectious disease patient care, but also for combatting the spread of antibiotic resistance^1–4^. Implementing the most appropriate clinical care requires rapid and accurate diagnostic information^5^.

Phenotypic AST tests the ability of antibiotics to inhibit bacterial growth, thereby providing key actionable information to physicians to determine proper antibiotic therapies^6^. This test is most commonly accomplished by a variation of a broth microdilution assay (BMD), a method that determines minimum inhibitory concentrations (MICs) for each tested antibiotic for a bacterial isolate derived from a patient sample^7^. To determine an accurate MIC for a given antibiotic, a range of concentrations must be tested. Thus, AST “panels” comprise multiple antibiotics, each tested at multiple concentrations^6^.

Phenotypic AST requires determining the difference between bacterial growth and the lack thereof, thereby limiting the speed with which results can be obtained. The Clinical and Laboratory Standards Institute (CLSI) BMD reference method, the “gold standard” phenotypic AST method, requires an incubation of 16–24 hours^7^. After this incubation period, results can be read visually. Some automated AST platforms enable faster reporting by using sensitive optical readers or including metabolic probes during incubation to visualize bacteria earlier^6,8^. These systems periodically monitor (every 15–30 minutes) every well of each panel and can report results for some strains in as few as 4–8 hours. However, in practice, the platforms have reporting modes of 9–13 hours, and average times-to-result of 7–16 hours, rendering them overnight assays in many clinical laboratory workflows^6,8,9^. Furthermore, the presence of metabolic probes may compromise measurement accuracies by interfering with antibiotic-bacterium interactions^10^. Finally, because these tests require periodic monitoring of every well on a test panel, introducing a new antibiotic necessitates removal of a different antibiotic or reduces the throughput of the overall system.

The key to performing “same-shift” (<6-hour) AST lies in developing an accurate and sensitive bacterial amplification method that does not affect cell growth and is able to account for shape-modifying growth modes that can be induced by antibiotics^8^. In addition to killing the organisms or preventing their replication, antibiotics can cause significant changes in bacterial cell morphology prior to cell death. For example, upon binding to penicillin binding proteins (PBPs), beta-lactam antibiotics can induce temporary bacterial elongation (filamentation) or swelling (protoplast or spheroplast) at antibiotic concentrations above MICs^11^. In these growth modes, bacteria enlarge but do not septate^12,13^. As a result, current automated systems, which measure bacterial density or metabolic activity, cannot accurately differentiate when bacterial cells are expanding in size versus dividing, and therefore cannot report results in <6 hours^6^. Beta-lactams as a class account for 70% of US antibiotics prescriptions; thus, it is imperative for a useful AST method to accurately capture their activity^13^.

To elucidate bacterial morphologies, academic and industrial researchers have recently developed multiple novel approaches that enable individual bacteria to be interrogated^8^. These include high-power optical microscopy^14^, micromachined cantilevers^15^, microfluidic approaches^16^, and flow cytometry^17^. Although powerful technologies, the complexities of these platforms limit throughputs, increase per-test costs, and decrease the number of antibiotics that can be tested in parallel^8^. Furthermore, by drastically decreasing the number of bacteria tested per antibiotic concentration, these methods may be prone to statistical sampling errors.

Here, we introduce a novel approach that allows phenotypic AST determinations for non-fastidious bacteria within 5 hours. After a 4-hour incubation in cation-adjusted Mueller-Hinton broth (CAMHB) and antibiotic^7^, this assay measures bacterial concentrations by binding a universal small-molecule amplifier to bacterial surfaces. Surface binding enables the method to account for bacterial morphological changes in response to antibiotics. Because this technique requires only a single, endpoint read, it should also enable larger antibiotic menus to be run for each patient sample.

## Results

**Figure 1:**
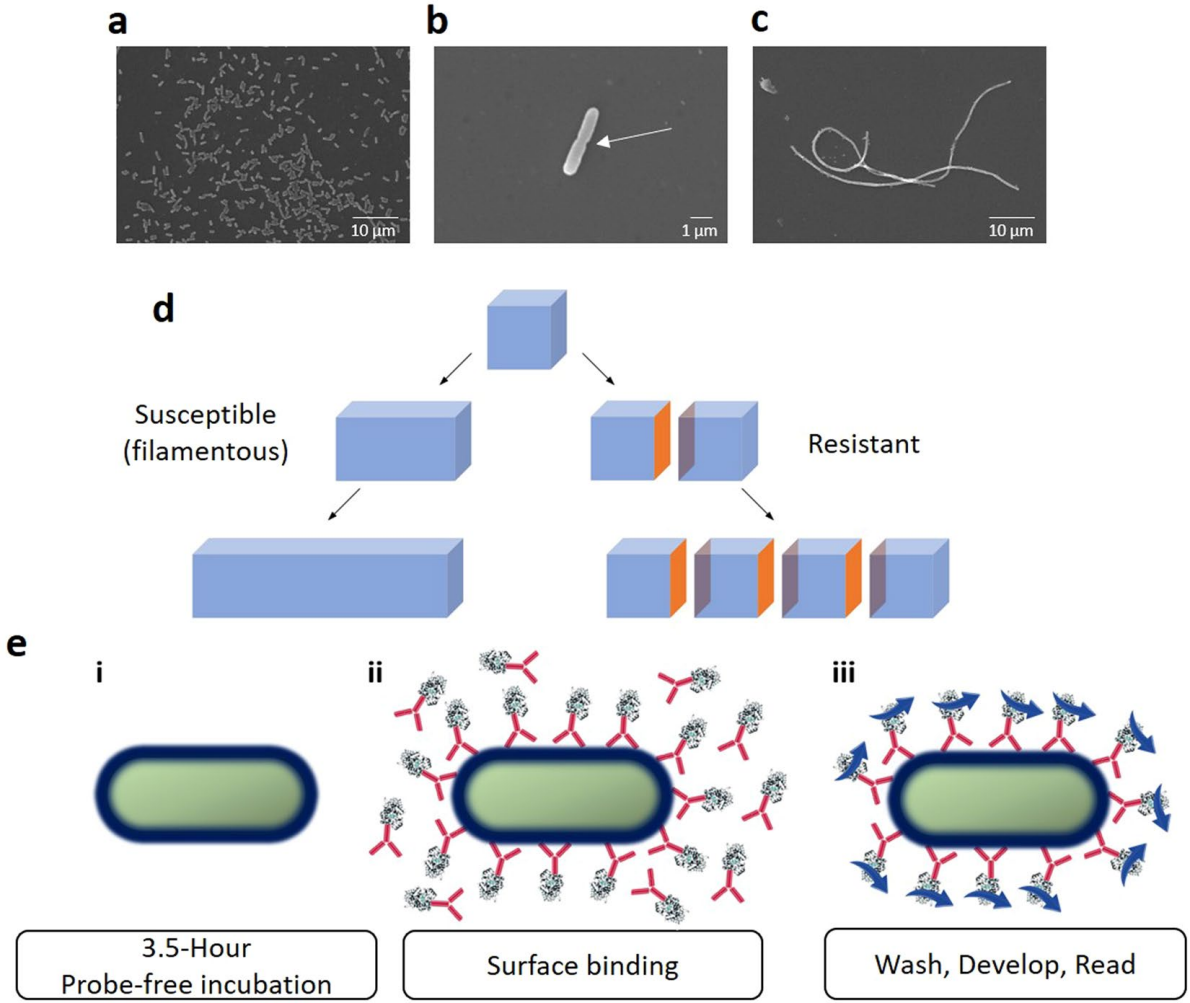
Surface area assay importance and design. (**a**–**c**) Scanning electron micrograph of *E. coli* grown on tryptic soy agar plates containing 5% sheep blood (**a**) or for 4 hours in cation-adjusted Mueller-Hinton broth (CAMHB) without (**b**) or with (**c**) an inhibitory concentration of aztreonam. (**a**,**c**) Magnification = 2,000×; (**b**) Magnification = 10,000×. (**d**) Schematic representing a bacterium undergoing two replication cycles under filament-inducing (left) or normal growth (right) conditions. Available surface area unique to septating bacterial cells is marked in orange. (**e**) Schematic and flowchart of the proof-of-principle surface-binding assay illustrating (i) probe-free growth, (ii) antibody-HRP binding, and (iii) signal development after unbound probes are removed. The signal development steps are only necessary when HRP is used for amplification.

Performing <6-hour phenotypic AST requires a method capable of distinguishing changes in bacterial morphology^11–13^. For example, since cells that initially filament and eventually die in the presence of beta-lactams are susceptible – the antibiotic is effectively binding PBPs – filamented bacterial cells in antibiotic concentrations at and above the MIC must be read as “susceptible.” Meanwhile, the smaller, truly resistant cells above the MIC must be read as “resistant”^11–13^. The optical micrographs in Fig. 1a–c show aztreonam-susceptible *Escherichia coli* cells in media containing no antibiotic or aztreonam at a concentration greater than the MIC after 4 hours of treatment with the antibiotic. Bacterial cells cultured in the absence of aztreonam (Fig. 1a,b) are drastically shorter than the filaments formed in the presence of aztreonam (Fig. 1c) and demonstrate evidence of active replication and septation (Fig. 1b). The *E*. *coli* filaments observed in the susceptible cells can persist for 6–8 hours after aztreonam is introduced, thus waiting for cell lysis is not an option if same-shift results are to be obtained.

The foundation of the assay introduced here is that measurements of bacterial surface area enable delineation of different bacterial morphologies. Standard cellular assays – viability assays or assays for ATP, NADH, enzymes, or nucleic acids – serve as proxies for bacterial metabolism and/or cellular volumes^18^. Thus, after the equivalent of two doublings, they cannot differentiate between four true daughter microbes and a filament comprising four non-septated cells, illustrated in the schematic in Fig. 1d^12^. In contrast, surface area measurements enable differentiation: the lack of septation yields a reduced surface area for the filamented microorganisms compared with resistant populations, whereas truly dividing cells introduce new surface area with each septation event, marked in orange in Fig. 1d. The difference is further pronounced in cells that undergo PBP binding-induced swelling, in which replication ceases. For antibiotics of other classes that do not bind PBPs, surface area measurements also differentiate replicating from inhibited bacteria based on the number of cells in each well.

For initial feasibility studies to determine whether bacterial detection by surface-binding could provide meaningful antibiotic susceptibility data, immunoglobulin G (IgG) antibodies for whole bacterial antigens were selected as surface-binding probes^19^. The large, 150 kDa antibodies were selected to ensure the probes would remain external to the bacteria. The assay is schematically illustrated in Fig. 1e. Following a 3.5-hour incubation period in which no probes are present (i), surface-binding antibody-horseradish peroxidase (HRP) conjugates are introduced (ii)^20^. After a short incubation to allow antibodies to attach to the bacterial surfaces, unbound antibodies are removed from the bacterial sample, followed by optical signal development with HRP (iii)^20^. An “endpoint” optical read is then performed using a standard microplate reader.

**Figure 2:**
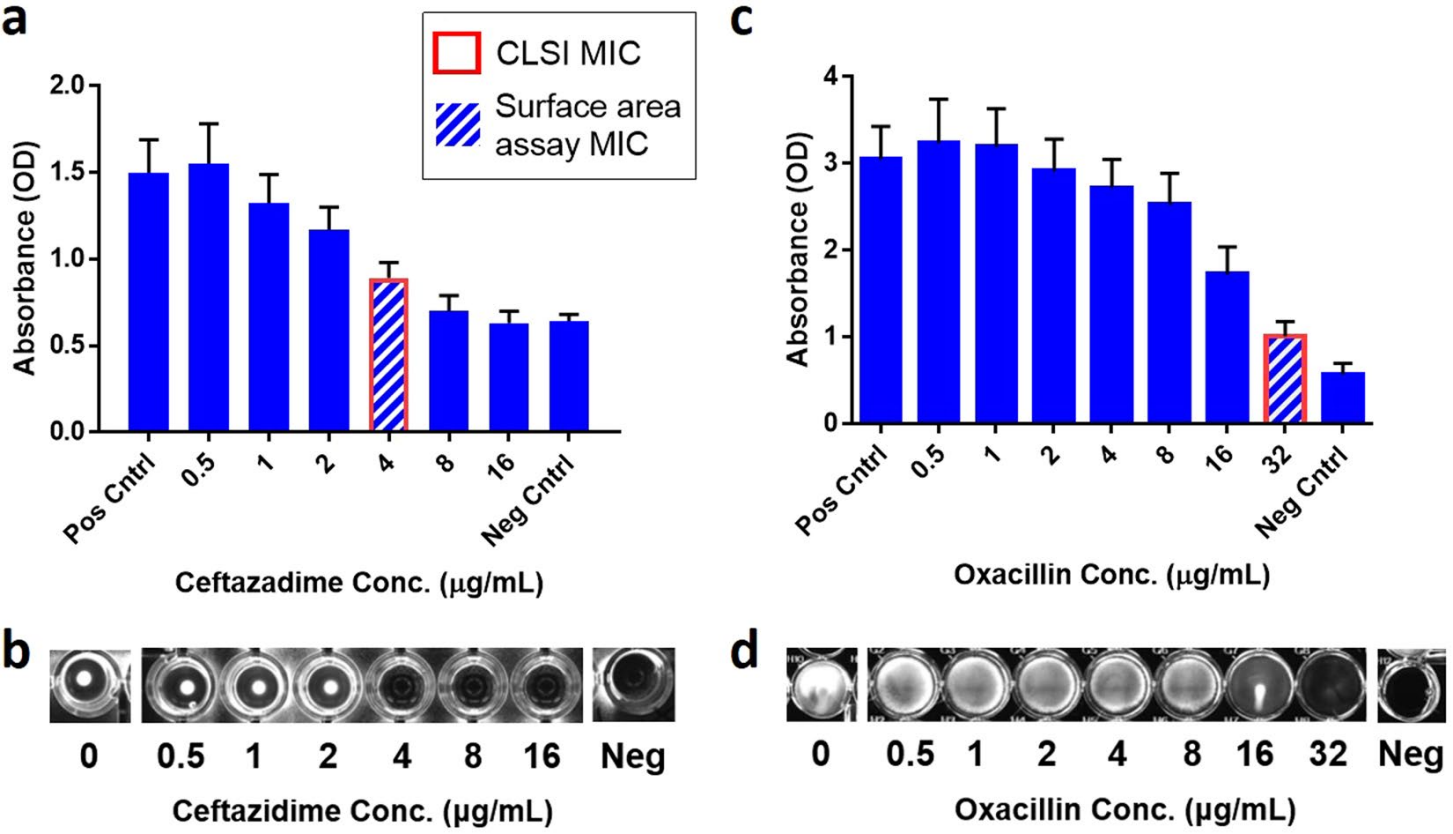
Surface area assay proof-of-principle using antibody probes. (**a**) Surface area binding assay data for a clinical *E*. *coli* sample treated with serially diluted concentrations of ceftazidime after 3.5 hours of growth. The assay gives an MIC of 4 μg/mL, shown by a hashed bar, which agrees with the CLSI reference method MIC, highlighted in red. Ceftazidime is known to induce filamentous growth in susceptible *E*. *coli* samples. (**b**) Optical picture of the 96-well plate in which the CLSI reference method was processed after 20 hours of bacterial growth. (**c**) Surface area binding assay data for a clinical *S*. *aureus* sample treated with serially diluted concentrations of oxacillin after 3.5 hours of growth. The assay gives an MIC of 32 μg/mL, shown by a hashed bar, which agrees with the CLSI reference method MIC, highlighted in red. (**d**) Optical picture of the 96-well plate in which the CLSI reference method was processed after 16 hours of bacterial growth. In panels (**b**) and (**d**) experiments were repeated in triplicate and error bars represent the standard deviation. Additionally, Pos Cntrl = no antibiotics present and Neg Cntrl = inoculated saline.

Antibody-HRP probes are found to yield signals proportional to bacterial concentrations. Results with filament-inducing ceftazidime and protoplast-inducing oxacillin for clinical *E*. *coli* and *Staphylococcus aureus* samples, respectively, are shown in Fig. 2. Because the surface area of susceptible filaments is reduced compared to truly resistant, dividing bacteria, repeated tests showed the MIC for filament-inducing antibiotics should be the lowest antibiotic concentration with a signal intensity ≤2/3 that of the positive growth control. This yields an MIC of 4 μg/mL in Fig. 2a, matching the MIC obtained from the CLSI reference method, Fig. 2b. Although protoplasts can swell significantly, and thereby increase surface area, bacteria in this growth mode cannot replicate. Empiric studies determined that the signal cutoff for growth in this case should be ≤1/2 that of the positive control signal. This yields an MIC value of 32 μg/mL in Fig. 2c, matching the CLSI reference method MIC, Fig. 2d. Taken together, these data demonstrate that surface area binding enables accurate MIC determinations for filament-inducing and protoplast-inducing antibiotics in gram-negative and gram-positive bacteria, respectively.

Reliance on antibody-based binding for AST is not ideal because it would likely necessitate large cocktails of polyclonal antibodies to achieve effective binding across all strains and species of bacteria^21,22^. In our *S*. *aureus* test case, for example, enhanced monoclonal antibody binding was likely achieved due to this species’ expression of Protein A, which binds the conserved fragments of IgGs on its surface^23^. The high affinity of Protein A to IgGs increased total antibody binding to bacterial surfaces, resulting in a positive-to-negative control ratio >5 (hereafter termed “dynamic range”), Fig. 2c. In contrast, inconsistent antibody binding was observed for different isolates of gram-negative bacteria, such as *E*. *coli*, likely owing to their highly-varied membranes^21^. Furthermore, the dynamic ranges were often <2, Fig. 2a.

**Figure 3:**
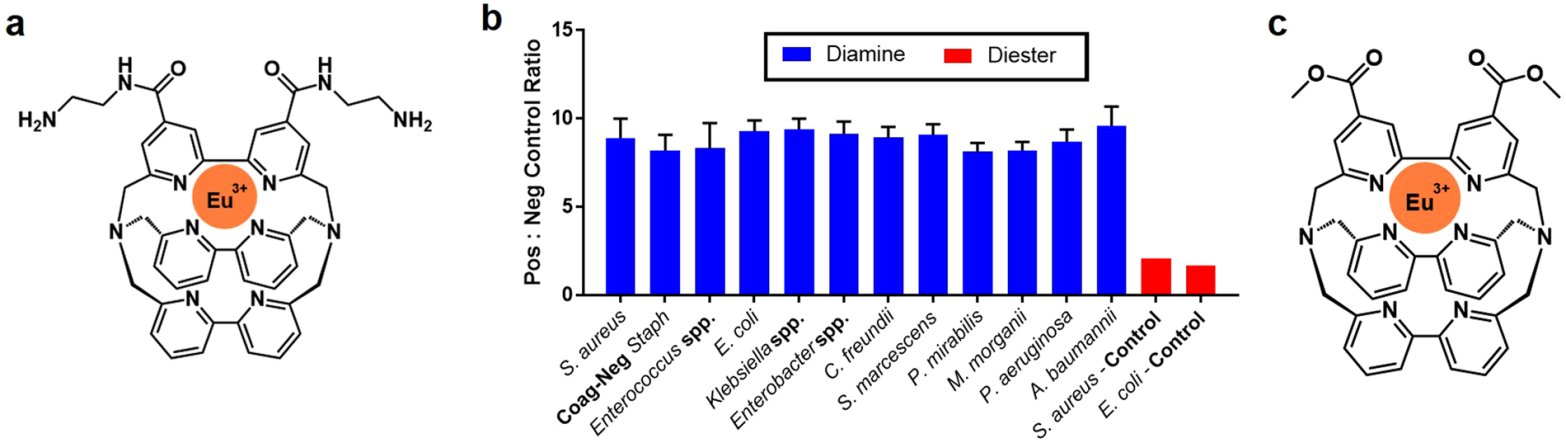
Bacterial surface binding performance of Eu-cryptate probes. (**a**) Eu-cryptate-diamine structure. (**b**) Surface area assay signal ratio of positive-to-negative controls for 13 species/genera of non-fastidious bacteria for Eu-cryptate-diamine or Eu-crypatate-diester probes. Positive controls were 4-hour growth in MHB and negative controls 4-hour growth in saline. Each species/genera data for the Eu-cryptate-diamine represents the ratio average ±1 standard deviation of a minimum of 20 individual isolates, each performed in triplicate. (**c**) Eu-cryptate-diester structure.

To overcome the inherent strain-to-strain and species-specific biochemical variability of bacteria, a charge-based chemical binding approach was developed. Bacterial membranes and walls have high anionic charges and rely on divalent cations for stability^22,24^. Thus, a cationic probe capable of binding bacterial surfaces and providing sufficient signal amplification to enable solution-phase optical measurements was sought. The known europium-cryptate-diamine (Eu-cryptate-diamine) chelate shown in Fig. 3a could fulfill both of these requirements^25^. The amines provide positive charge necessary for electrostatic binding to negative bacterial surfaces at neutral pH. The Eu-cryptate organometallic ligand exhibits intense time-resolved fluorescence (TRF) that enables this ~900 g/mol molecule to provide signal amplifications equivalent to the ~45 kDa HRP enzyme^20^. TRF can be read using a standard plate reader and requires no development step^25^, simplifying the assay workflow in Fig. 1e. Upon testing we found that the Eu-cryptate-diamine probe was indeed able to bind multiple genera and species of non-fastidious bacteria and consistently provided assay dynamic ranges >7.5, Fig. 3b. In order to confirm probe-bacteria interactions are electrostatic in nature, a neutral, Eu-cryptate-diester probe was tested, Fig. 3c. These amine-free probes demonstrated negligible binding to representative *S*. *aureus* and *E*. *coli* strains, Fig. 3b.

**Figure 4:**
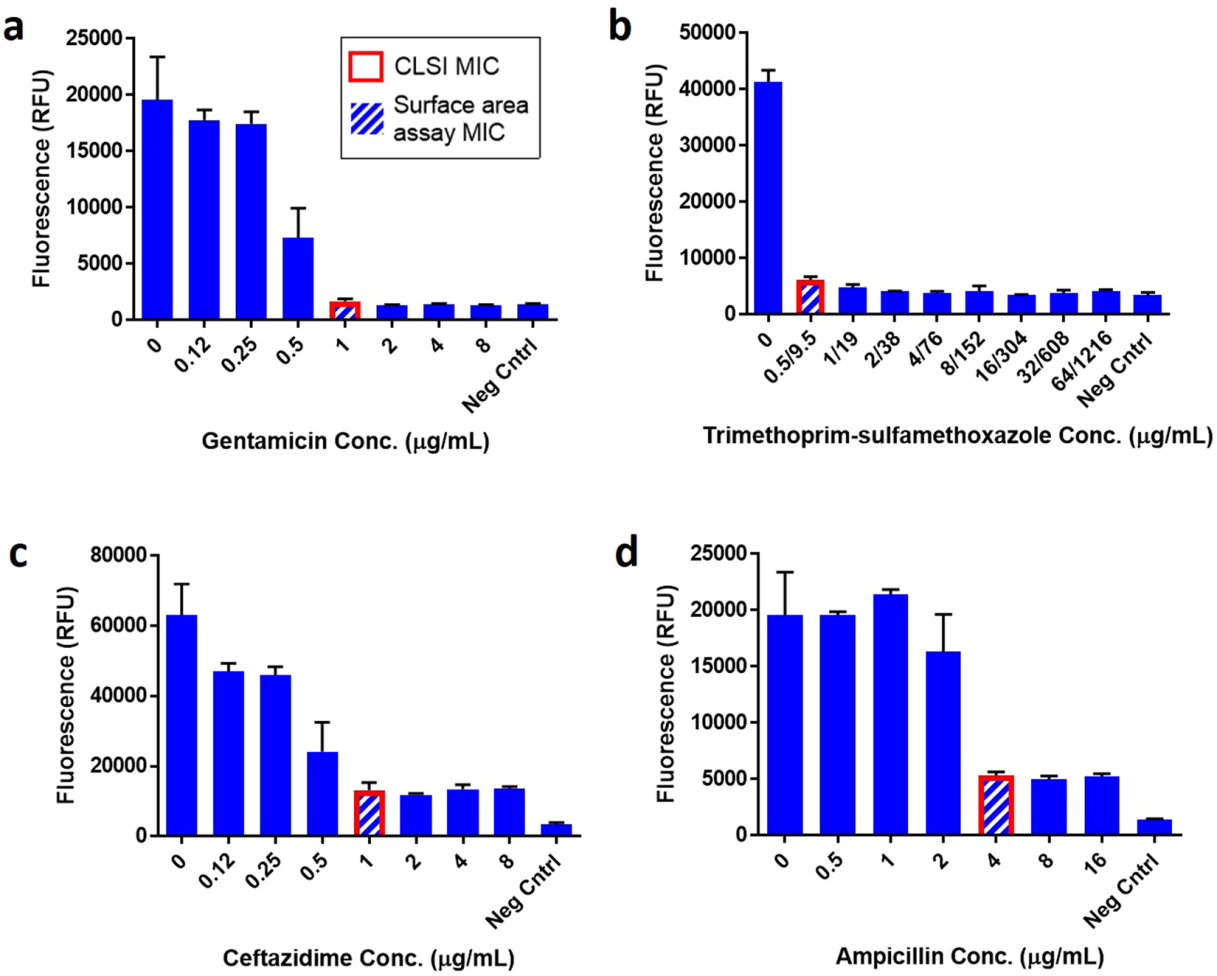
Eu-Cryptate-diamine probe assay results for complete dilution series of representative antibiotics. Data for clinical *E*. *coli* samples [SeLux Clinical Isolates 130 (**b**) 358 (**c**) 657 (**a**,**d**)] treated with (**a**) gentamicin, a bactericidal antibiotic; (**b**) trimethoprim-sulfamethoxazole, a bacteriostatic antibiotic; (**c**) ceftazidime, a filament-inducing antibiotic; and (**d**) ampicillin, a spheroplast-inducing antibiotic. In each panel the assay MIC is shown by a hashed bar and the CLSI reference method MIC is highlighted in red. Positive controls were 3.5-hour growth in MHB and negative controls 4-hour growth in saline. Data shown is the average of 3 replicates (2 replicates in positive control) ±1 standard deviation.

The Eu-cryptate-diamine probe was then tested for its ability to enable accurate MIC determinations for antibiotics that exhibit different effects on *E*. *coli* cells. Aminoglycosides, such as gentamicin, are bactericidal antibiotics that do not induce filamentation or swelling^13^. The MIC obtained by the surface area assay for this antibiotic agrees with the MIC determined by the CLSI reference method, Fig. 4a. Trimethoprim-sulfamethoxazole (SXT), a bacteriostatic antibiotic^13^, also shows clear MIC definition, Fig. 4b. The MIC again agrees with the CLSI reference value.

Similar accuracies are obtained for beta-lactam antibiotics. Beta-lactams, such as the cephalosporin ceftazidime^13^, show relatively higher backgrounds in wells at and above the MIC, likely due to bacterial filamentation. Here, the MIC value of 1 μg/mL agrees with the CLSI reference result, Fig. 4c, and the signal trend is similar to that observed in Fig. 2a, suggesting that the Eu-cryptate is also binding bacterial surfaces. Another beta-lactam, ampicillin, which induces spheroplast formation (swelling) in gram-negative organisms^13^, also shows relatively higher background signal in wells at and greater than the MIC, Fig. 4d, similar to that observed in Fig. 2c. The MIC chosen was the lowest dilution with a signal ≤1/2 that of the positive control, 4 μg/mL, which again agrees with the CLSI reference result.

**Figure 5:**
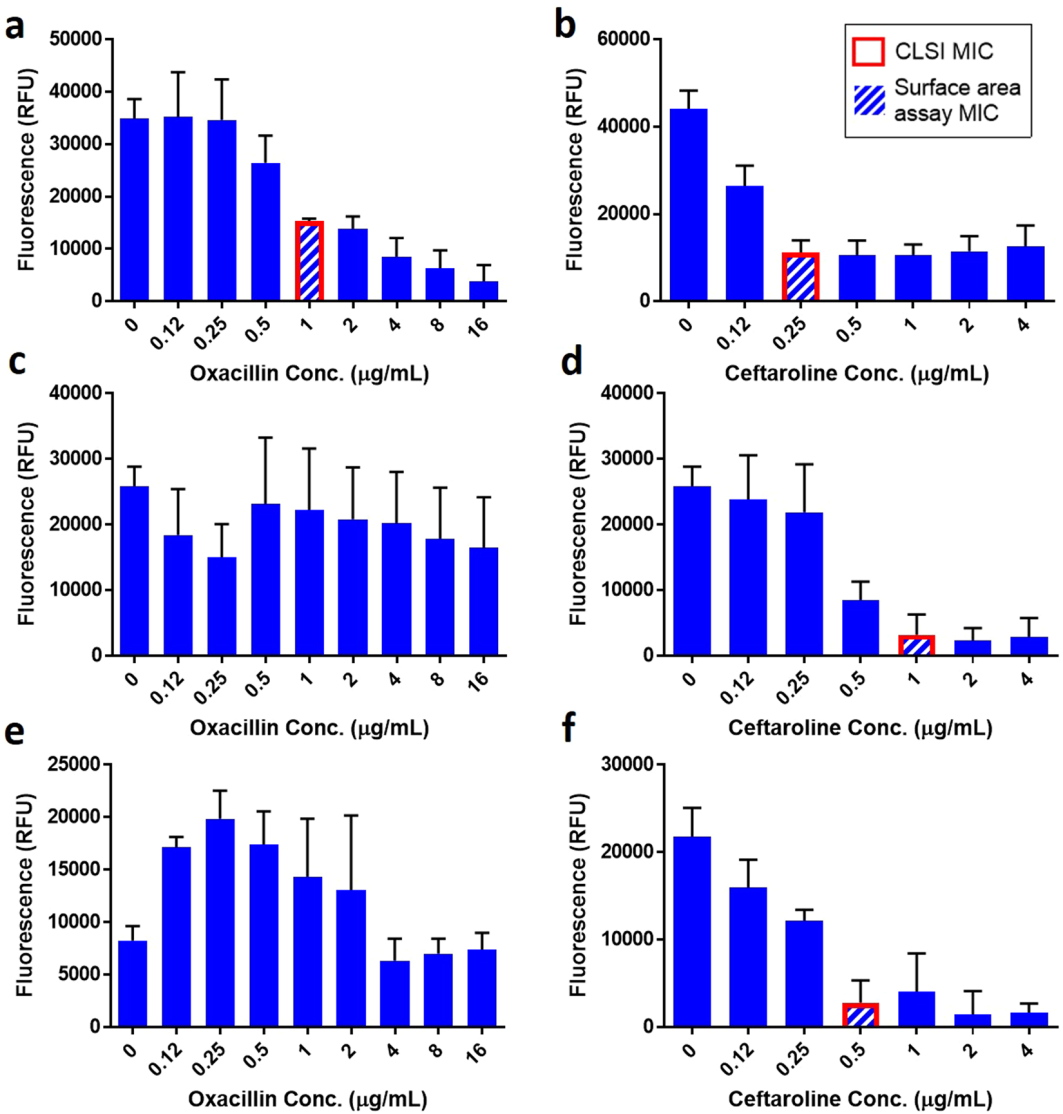
Eu-Cryptate-diamine probe assay results for MSSA and MRSA. Data for *S. aureus* isolates: CDC/FDA AR bank #569 (**a**,**b**), BEI Resources BR15 (**c**,**d**), and CDC/FDA AR bank #227 (**e**,**f**) treated with oxacillin (**a**,**c**,**e**) and ceftaroline (**b**,**d**,**f**). In each panel the assay MIC is shown by a hashed bar and the CLSI reference method MIC is highlighted in red. If the MIC is greater than the tested range (**c**,**e**), no MIC is indicated. Surface assay MICs were determined as the lowest concentration of antibiotic at which signal is ≤50% (oxacillin) or ≤33% (ceftaroline) of the positive control (background subtracted). Data shown is the background-subtracted average of 3 replicates ±1 standard deviation. Background is defined as the signal present in uninoculated wells.

The surface binding assay presented here provides a unique opportunity to discern phenotypic MICs for gram-negative bacteria in beta-lactam antibiotics, where filamentation can potentially skew results. Beta-lactams are also commonly used against gram-postive organisms. Therefore, we assessed the utility of the surface binding assay to predict MICs in three isolates of *S*. *aureus* in two relevant beta-lactam antibiotics, Fig. 5. Here, the surface-binding assay accurately predicted oxacillin sensitivity in a methicillin-sensitive *S*. *aureus* (MSSA) isolate, which had an MIC of 1 μg/mL in the reference method Fig. 5a, and oxacillin resistance in two methicillin-resistant *S*. *aureus* (MRSA) isolates, which had MICs of ≥32 μg/mL Fig. 5c,e. The surface-binding assay also accurately determined MICs for ceftaroline, another beta lactam antibiotic, for each of these three *S*. *aureus* isolates. These data demonstrate the assay’s ability to correctly identify ceftaroline susceptibility in oxacillin-resistant MRSA strains and, thus, accurately determine MICs independently for each drug.

**Figure 6:**
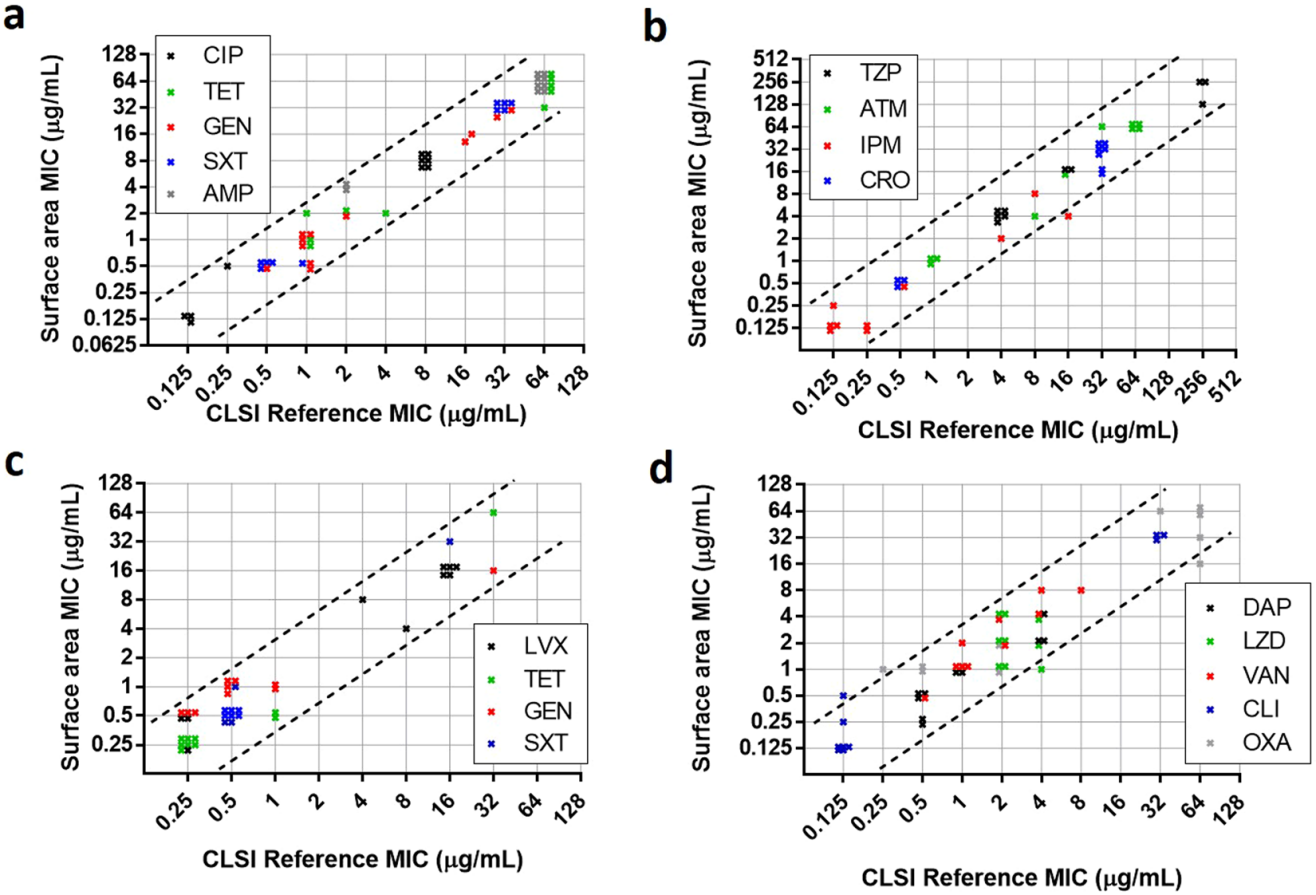
Rapid surface area assay vs. CLSI-determined MICs across representative antibiotics from each of 13 primary antibiotic classes. Plots of surface area assay-derived MICs vs. CLSI reference method MICs for 10 *E*. *coli* samples tested with (**a**) 5 broad-spectrum antibiotics and (**b**) 4 Gram-negative-specific antibiotics and 10 *S*. *aureus* samples tested with (**c**) 4 broad-spectrum antibiotics and (**d**) 5 Gram-positive-specific antibiotics. Each bug-drug MIC is shown by a single “x,” with datapoints shifted where necessary to enable visualization of every individual datapoint. The space between the dashed lines on each plot demonstrates FDA “essential agreement,” ±1 dilution from the CLSI reference method result. For each species, 5 samples were clinical isolates and 5 were FDA-CDC “challenge” samples. Broad-spectrum classes are aminoglycosides (gentamicin, GEN), fluoroquinolones (ciprofloxacin, CIP), penicillins (ampicillin, AMP), sulfonamides (trimethoprim-sulfamethoxazole, SXT), and tetracyclines (TET). Gram-negative-specific antibiotics are beta-lactam/beta-lactamase inhibitor combinations (piperacillin-tazobactam, TZP), carbapenems (imipenem, IPM), cephalosporins (ceftazidime, CAZ), and monobactams (aztreonam, ATM). Gram-positive-specific antibiotics are lipopeptides (daptomycin, DAP), glycopeptides (vancomycin, VAN), penicillins (oxacillin, OXA), and oxazolidinones (linezolid, LZD). Details for the deterimination of the surface area assay MICs and antibiotic ranges tested are included in the supplementary information.

To provide clinical utility, an AST method must give accurate results for all antibiotic classes and across strains with varied resistance profiles and distinct resistance mechanisms. Ten isolates each of the two most common clinical pathogens, *E*. *coli* and *S*. *aureus*, were tested against 9 antibiotics each, representative of different major antibiotic classes. Five isolates of each species were standard clinical isolates and the other five were unique “challenge” strains obtained from the CDC-FDA Antibiotic Resistance Isolate Bank^26^. The data in Fig. 6 compare MICs determined using the data analysis steps described above for the 5-hour surface-binding method with those determined by the overnight CLSI reference method. These data demonstrate good agreement across all antibiotic classes, MIC ranges, and resistance mechanisms tested (Tables S1, S2), with fewer than 3% of results falling outside of essential agreement, defined by the FDA as an MIC within ±1 dilution to the CLSI reference method. Of the results that did not meet essential agreement, all met categorical agreement, defined by the FDA as accurate sensitivity or resistance results based on clinical breakpoints. These data demonstrate that the surface area assay presented here is versatile and can be effectively used to accurately predict MICs across a wide variety of antibiotics in gram-negative and gram-positive organisms featuring a diverse array of resistance mechanisms.

## Discussion

This assay leverages electrostatic binding of a highly fluorescent molecule to bacterial surfaces to predict antibiotic susceptibility. Though this species-agnostic molecule binds all bacterial species tested, it is important to note that only a subset of non-fastidious species have been tested to date, Fig. 3b. Further testing will be required to determine if there are limitations binding other non-fastidious and fastidious species and whether the approach is feasible for fungi. In particular, limitations in binding may be expected where nutrient supplements, such as lysed horse blood, are required for microorganism growth.

The method described here is able to rapidly determine antibiotic susceptibility in diverse types of antibiotics for two of the most prevalent bacterial pathogens, *E*. *coli* and *S*. *aureus*. In particular, susceptibilites to antibiotics that cause filamentation and swelling are detectable before lysis by this method. Because the surface area assay relies on interaction of a detector molecule with the surface of bacteria, it is a possible concern that antibiotics that target the cell surfaces might cause unreliable data. However, testing with multiple organisms across mutiple surface-targeting antibiotics, such as beta-lactams and vancomycin, demonstrated the broad reliability of the method.

The assay was able to accurately predict MICs to a suite of surface-targeting antibiotics in both sensitive and multidrug resistant *E*. *coli* and *S*. *aureus* organisms with a wide variety of antibiotic resistance mechanisms. Though this study attempted to cover multiple resistance mechaisms by using both clinical and FDA-CDC challenge panel samples, it is important to note that the small number of samples tested in this study fall well short of exhibiting all potential resistance mechanisms. For example, the method’s ability to accurately detect efflux pump-mediated resistance, a key mechanism of *Pseudomonas aeruginosa*, has not been tested. Future studies comprising greater numbers of samples and species are thus required.

The assay presented here may offer a powerful yet simple approach for performing rapid phenotypic AST determinations. One of the benefits of this assay is that, as an endpoint assay, it does not require periodic monitoring of bacterial growth in all conditions to accurately predict MICs. However, since non-fasitious bacteria do not all grow at the same rate – for example, *Enterococcus* spp. and *P*. *aeruginosa* are known to have slower doubling rates than *S*. *aureus* and *E*. *coli* – it may be necessary to ensure sufficient growth has been achieved before initiating the endpoint assay described in this work. The surface area assay’s compatibility with microplates and standard automated hardware components, such as plate readers and liquid handlers, should enable high assay throughputs at low costs. Taken together, these features should enable the use of high-density microplates, such as 384-well plates, potentially allowing more antibiotics to be tested in parallel for each patient sample.

By speeding the reporting of AST results and expanding the antibiotics that can be tested, this platform should enable hospitals to simultaneously improve patient care, decrease lengths of stay, and meet antibiotic stewardship goals. Furthermore, the compatibility of this endpoint assay with 384-well microplates, which provide ample space for incorporation of new antibiotics, should transform the speed with which newly approved antibiotics enter the clinic and may even speed antibiotic development.

## Methods

### Bacterial culturing and media

Bacterial challenge isolates were obtained from the CDC and FDA Antibiotic Resistance Isolate Bank and BEI resources. Other bacterial isolates were purchased from TriCore Reference Laboratories and the ATCC. Additional bacterial isolates were kind gifts from American Esoteric Laboratories and Sunrise Medical Laboratories. Complete information about the bacterial isolates used in this study is included in the supplementary information. All isolates were cultured on tryptic soy agar with 5% sheep’s blood (Northeast Laboratory) prior to testing.

### CLSI Broth microdilution reference method

The broth microdilution reference method was run according to the CLSI guidelines^7^. Each strain was tested on frozen, 96-well antibiotic plates (custom order, International Health Management Associates, Inc., IHMA or Thermo Fisher Scientific), with the appropriate antibiotics depending on its classification. Bacterial inoculums were made to a 0.5 McFarland, diluted, and then inoculated in each well except the negative control, to achieve approximately 5 × 10^5^ CFU/mL final concentration in each well. Sterile lids were then placed on the top of each plate before being placed in a 35 °C ambient air incubator for 16–20 hours or when applicable, plates were returned to the 35 °C ambient air incubator for additional time and reexamined at 24 hours after initial inoculation. The plates were read against a backlight to determine the strain’s minimum inhibitory concentration (MIC). Exceptions to reading complete inhibition of growth were made based on CLSI guidelines. Plates were photographed with a darkfield microplate camera (custom-developed hardware).

### Proof of concept antibody probe surface area assay

Thawed, frozen panels from IHMA containing antibiotics and cation-adjusted Mueller-Hinton Broth (CAMHB) were used. Using direct colony suspension method, several colonies of bacteria from a fresh blood agar plate (16–18 hours) were suspended in 0.85% saline (Northeast Laboratories) to achieve a standardized inoculum of 0.5 McFarland (1–2 × 10^8^ CFU/mL). Each well except the negative control well was inoculated to achieve approximately 5 × 10^5^ CFU/mL. The plates were placed in a shaking incubator of 150RPM at 35 °C for three hours and 45 minutes.

After incubation, 25 μL of a magnetic particle solution (magnetic silica particles, SiMAG-Q (50 mg/mL, Chemicell, Berlin, Germany) suspended in 2× Borate Buffer (Thermo Fisher Scientific, final concentration: 15.63 μg/mL) was added, followed by 25 μL of the *S*. *aureus* or *E*. *coli* antibody-HRP conjugate solution (polyclonal HRP-conjugate (*S*. *aureus*: Fitzgerald or *E*. *coli*: Abcam) in 5× ELISA/ELISPOT buffer (eBioscience), final concentration, 1.25 μg/mL). The plates were then placed on a shaker at 450RPM for 20 minutes to allow binding of magnetic particles and antibody-HRP to the bacteria.

Following the binding step, the “Crocodile 5-in-one” machine (ELISA mini Workstation, Titertek Berthold) was outfitted with a custom magnet array insert and used to automate magnetic capture and washing. The microplates were washed thrice with wash buffer at 250 μL/well (Phosphate Buffer Solution with Tween 20, PBST) to remove unbound antibody-HRP. A chromogenic substrate solution,1 × TMB (3,3′,5,5′-Tetramethylbenzidine, eBioscience) was added at 100 μL/well and placed back on the shaker for 20 minutes. Once done, 50 μL of sulphuric acid (1 M) was added to stop the reaction mixture. Absorbance was read at 450 nm.

### Eu-Cryptate probe surface area assay

For each strain, frozen antibiotic panels from IHMA or Thermo Fisher (SensiTitre) were used. Bacterial inoculums were made to a 0.5 McFarland, diluted, and inoculated in each well except the negative control, to achieve approximately 5 × 10^5^ CFU/mL. Sterile lids were placed on top of the plates and placed in the shaking incubator at 150RPM and 35 °C for four hours. After incubation, 100 μL of a detergent solution (either Phosphate-Buffered Saline with 1% Tween-20 (PBST, Sigma-Aldrich) or 0.03% Cetyltrimethylammonium bromide with 0.1 M EDTA, (Sigma-Aldrich)) was added to each well. The plates were placed on a shaker at 450RPM for 10 minutes, followed by centrifugation at 2500 × *g* for 2.5 minutes. The wells were aspirated with the MultiFlo FX multi-mode dispenser (BioTek) and 100 μL 1 × PBST (G-Biosciences) plus Eu-cryptate-diamine (20 ng/well, CisBio, USA) was added back into the wells. The plates were shaken for 10 minutes. The plates were then centrifuged, the wells aspirated, and 200 μL 1 × PBST were added to each well. These steps were repeated twice. After the final wash, time-resolved fluorescence (TRF) for all wells was read at 330/615 nm.

For surface area binding experiments with the Eu-cryptate-diamine and the Eu-cryptate-diester in Fig. 3, the process described above was followed. All species of bacteria were initially grown in CAMHB (Becton Dickinson) and the Eu-cryptate-diester or Eu-cryptate-diamine was used for binding. The Eu-cryptate-diester synthesis and europium insertion was performed similarly to the published Eu-cryptate-diamine synthesis^27^.

### Microscopy Process (SEM)

Microscopy was performed on *E*. *coli 25922* grown in CAMHB, CAMHB with aztreonam (64 μg/mL), and from a colony harvested from bacteria grown on tryptic soy agar plates containing 5% sheep blood. A drop of both broth cultures was transferred to a slide and heat-fixed over a gentle flame. Culture from direct colony was smeared onto a loopful of water on the slide, then heat-fixed over the flame. Crystal violet stain (Sigma-Aldrich) was added to the fixed culture and allowed to stand for 60 seconds. The stain was washed off and Lugol solution (Sigma-Aldrich) was added to the smear for another 60 seconds. After iodine was washed off, decolorizer was added to the smear for 30 seconds before being washed off, then counterstained with safranin (Sigma-Aldrich) for 60 seconds. The stained *E*. *coli* 25922 were sputter-coated with gold-palladium for 45 seconds (Denton Desk IV, Moorestown, NJ) and observed with a scanning electron microscope (JEOL 6390, JEOL USA, Peabody, MA) at an accelerating voltage of 10 kV.

## Electronic supplementary material

Supplementary information

Supplementary Table 5

## Data Availability

All data generated or analysed during this study are included in this published article and its Supplementary Information file.
