# Supplementary material for: Microplate-based surface area assay for rapid phenotypic antibiotic susceptibility testing
Source: Sci Rep. 2019 Jan 18;9:237. doi: 10.1038/s41598-018-35916-0 (PMC6338723; doi:10.1038/s41598-018-35916-0)
Supplement: Supplementary file 1 — Supplementary information [file 41598_2018_35916_MOESM1_ESM.docx]

**Supplementary Information**

**Title:** Microplate-based surface area assay for rapid phenotypic antibiotic susceptibility testing

**Authors:** Kelly Flentie*, Benjamin Spears*, Felicia Giok, Nathan Purmort, Kayla DaPonte, Emma Viveiros, Nicholas Phelan, Cicely Krebill, Alec N. Flyer, David Hooper, David L. Smalley, Mary Jane Ferraro, Aleksandar Vacic, Eric Stern

**Table S1: *E. coli* isolates tested in Figure 6**

**
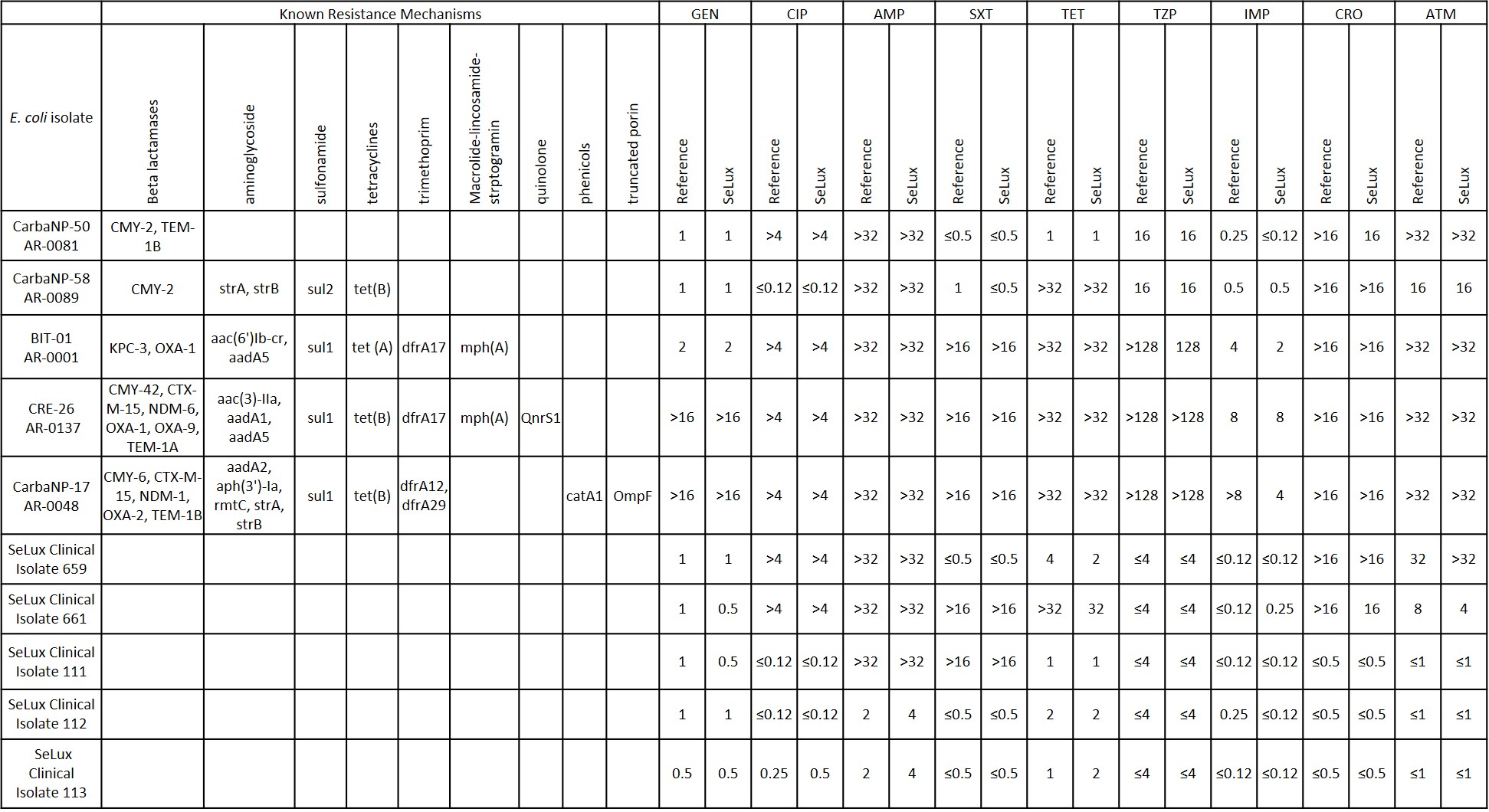
**

**Table S2: *S. aureus* isolates tested in Figure 6**

**
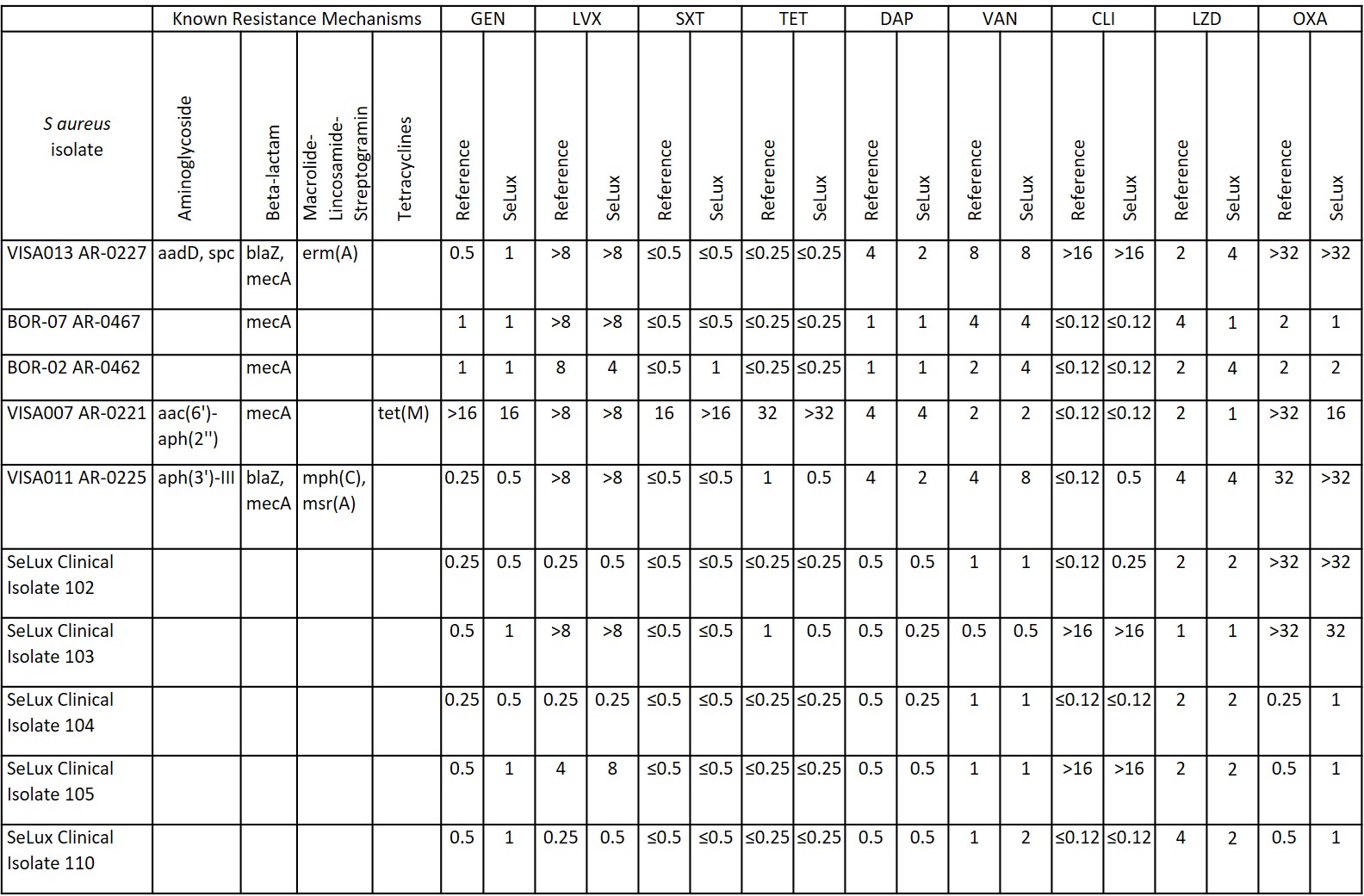
**

**Tables S1 and S2 References:**

1. CLSI. *Performance standards for antimicrobial susceptibility testing*. 28th ed. CLSI supplement M100. Wayne, PA: Clinical and Laboratory Standards Institute; 2018.
2. CLSI. *Methods for dilution antimicrobial susceptibility tests for bacteria that grow aerobically*. 11th ed. CLSI supplement M07. Wayne, PA: Clinical and Laboratory Standards Institute; 2018.
3. CDC and FDA Antibiotic Resistance Isolate Bank. Atlanta (GA): CDC. (2018).

**Surface area assay MIC determination rules:**

Tests were performed on multiple strains of each species of bacteria, *E. coli* and *S. aureus*, to determine MIC determination rules for each bacteria-antibiotic combination. **Tables S1** and **S2** detail the MIC determination rules for each combination that were used to produce the data shown in **Figure 6**.

**Table S3: *E. coli* antibiotic testing ranges and MIC deteriminations**

| **Antibiotic** | **Dilution Range (µg/ml)** | **MIC rule** |
| --- | --- | --- |
| GEN | 0.06-16 | MIC = first dilution ≤33% of positive control |
| CIP | 0.12-4 | MIC = first dilution ≤33% of positive control |
| AMP | 0.25-32 | MIC = first dilution ≤50% of positive control |
| SXT | 0.5/9.5-16/304 | MIC = first dilution ≤50% of positive control |
| TET | 0.25-32 | MIC = first dilution ≤33% of positive control |
| TZP | 4-128 | MIC = first dilution* ≤20% of positive control* |
| IPM | 0.12-8 | MIC = first dilution ≤33% of positive control |
| CRO | 0.5-16 | MIC = second dilution* ≤20% of positive control*, MIC ≤ 0.5 if first dilution* ≤15% of positive control* |
| ATM | 1-32 | MIC = second dilution* ≤20% of positive control*, MIC ≤ 0.5 if first dilution* ≤15% of positive control* |

*****Background subtracted.

**Table S4: *S. aureus* antibiotic testing ranges and MIC deteriminations**

| **Antibiotic** | **Dilution Range (µg/ml)** | **MIC rule** |
| --- | --- | --- |
| GEN | 0.06-16 | MIC = first dilution* ≤75% of positive control* |
| LVX | 0.12-4 | MIC = first dilution* ≤60% of positive control* |
| SXT | 0.5/9.5-16/304 | MIC = first dilution* ≤85% of positive control* |
| TET | 0.25-32 | MIC = first dilution* ≤60% of positive control* |
| DAP | 0.12-4 | MIC = first dilution* ≤50% of positive control* |
| VAN | 0.25-32 | MIC = first dilution* ≤30% of positive control* |
| LNZ | 0.06-8 | MIC = first dilution* ≤45% of positive control* |
| CLI | 0.12-16 | MIC = first dilution* ≤35% of positive control* |
| OXA | 0.03-32 | MIC = first dilution* ≤50% of positive control* |

*****Background subtracted.
